# Treatment Challenges in Acute Minor Ischemic Stroke

**DOI:** 10.3389/fneur.2021.723637

**Published:** 2021-09-07

**Authors:** Diana Slawski, Jeremy J. Heit

**Affiliations:** ^1^Department of Neurology, Stanford Health Care, Stanford, CA, United States; ^2^Department of Neuroimaging and Neurointervention, Stanford Health Care, Stanford, CA, United States

**Keywords:** stroke, low NIHSS score, endovascular treatment, intravenous thrombolysis, minor stroke

## Abstract

Patients with acute ischemic stroke may present with minor neurologic deficits. Acute treatment decisions depend on the disability imposed by the symptoms along with radiographic features. The presence of disabling neurologic deficits warrants intravenous thrombolysis, but the indications for endovascular therapy are less defined. The degree of disability, presence of a large vessel occlusion with perfusion mismatch, and collateral circulation status may all be factors in selecting patients for endovascular treatment. Identification of patients who are at risk for neurologic deterioration is critical to preventing poor outcomes in this patient population.

## Background and Current Status of Minor Ischemic Stroke Treatment

Clinical trials have shown that acute ischemic stroke can be treated with intravenous (IV) thrombolysis (1, 2) and/or endovascular thrombectomy (ET) (3–5). Treatment with thrombolysis is not without risk, and it is the physician's task to determine whether the benefit of treatment outweighs that risk for each individual patient. This risk-benefit assessment is aided by clinical scales that measure neurologic impairment and non-invasive brain imaging studies. The National Institutes of Health Stroke Scale (NIHSS) is a 42-point measure of stroke severity that is used throughout the US and elsewhere. In research and clinical practice, the NIHSS score is used to help guide treatment decisions and prognosis. Patients with a higher NIHSS score generally have a larger ischemic territory and worse outcomes if left untreated (6). In addition to the NIHSS score, neuroimaging is another key factor in making treatment decisions for thrombolysis. For all stroke patients a non-contrast head computed tomography (CT) is required for treatment with thrombolysis in order to exclude the presence of an intracranial hemorrhage and a large established ischemic infarction. Advanced imaging, such as CT or magnetic resonance (MR) perfusion, is an additional tool for more complex cases such as delayed presentation from symptom onset.

The NIHSS score and imaging are key to identifying patients who may benefit from IV thrombolysis treatment, though there are instances where these tools may be insufficient. Patients who present with mild symptoms and a low NIHSS score are an important example of how these screening tools may fail. The concept of “minor stroke” has been defined as NIHSS scores ≤ 5 by the American Stroke Association, but the score alone may not account for the disability incurred by certain symptoms as will be discussed below in more detail. The early clinical trials for IV thrombolysis and ET generally excluded patients with mild symptoms or no measurable deficit on the NIHSS (1, 7, 8). The presence of mild symptoms is one of the most commonly-cited reasons for not administering alteplase (9). Approximately 34% of acute ischemic stroke patients presenting with low NIHSS scores are not treated with alteplase (10) and another 30% who are otherwise eligible for ET are also not treated (11). Furthermore, studies on the natural history of minor strokes with NIHSS ≤ 5 have demonstrated that 25% of patients will have residual disability at 3 months (12). These statistics illustrate how a significant minority of patients with mild stroke are considered ineligible for treatment despite the increased risk of poor outcome. There is a critical need for more data and better screening tools to identify which patients with minor stroke symptoms would benefit from treatment. In this review, we will discuss the challenges in acute minor ischemic stroke and future directions to improve patient care.

## Minor Ischemic Stroke and Intravenous Thrombolysis

Prior to IV thrombolysis with alteplase, there are both clinical and radiographic criteria that should be met for treatment eligibility. Official guidelines put forth by the American Stroke Association recommend that patients with disabling symptoms, regardless of stroke severity measured by the NIHSS, should be treated with IV thrombolysis if they meet other standard criteria (13). This recommendation supports the use of alteplase in patients with low NIHSS so long as disabling symptoms are present. A large meta-analysis by Emberson et al. (14) pooled data from major trials, including NINDS, ECASS and IST, and over 6,000 patients were included. While only 10% of these patients had minor strokes with NIHSS 0–4 and disabling symptoms, there was a demonstrable benefit of treatment with alteplase compared to placebo with an odds ratio of 1.48 (1.07–2.06, 95% CI) for good outcome. This study informed the guideline recommendations.

The presence of disabling symptoms is a key factor in patient selection for IV thrombolysis; however, there is no unified definition in the literature for what constitutes “disabling symptoms.” Even some clinical trials, such as ECASS III, did not specify this term in more detail (1). In our practice, patients with limb weakness, language impairment, vision impairment, and hemineglect are considered to have disabling symptoms that warrant treatment with IV thrombolysis. By contrast, there is more literature about what might be considered “non-disabling symptoms.” In the NINDS-TPA trial, the investigators specifically noted that pure sensory symptoms, isolated ataxia, isolated dysarthria, and isolated facial weakness could be considered minor and non-disabling. A subsequent study found that application of this definition, rather than a particular score on the NIHSS, may better identify minor stroke patients who could do well without IV thrombolysis (12). Overall, qualifying symptoms as disabling or non-disabling can help distinguish which patients with mild stroke severity should be treated.

The ASA guidelines comment that patients with mild stroke severity (NIHSS scores ≤ 5) and no disabling symptoms should not receive alteplase (13). Rather, patients with mild symptoms might benefit from less aggressive medical treatment, including aspirin administration. The PRISMS trial was a randomized trial that compared IV thrombolysis with alteplase to aspirin (15). PRISMS was halted early, but the results suggested that acute ischemic stroke patients with low NIHSS scores of 0–5 and no disabling symptoms are unlikely to gain benefit from treatment with alteplase compared to aspirin (15). More recently, the use of dual-antiplatelet therapy in minor ischemic stroke or transient ischemic attack has gained favor based on evidence from two clinical trials (16, 17). Patients with NIHSS scores of 0–3 had significantly reduced risk of recurrent stroke when treated with aspirin and clopidogrel, with the most benefit gained within the first 21 days (18).

Imaging plays a supportive role in screening for patients who would benefit from alteplase. Routine non-contrast head CT is required prior to treatment to exclude cerebral hemorrhage or a large territory cerebral infarction but is unlikely to alter the decision for thrombolysis in the way that the presence of disabling symptoms might. However, advanced imaging plays a larger role in patients with delayed presentation or unknown time of symptom onset. The EXTEND trial evaluated alteplase treatment in patients with evidence of a salvageable penumbra on cerebral perfusion imaging between 4.5 and 9 h from symptom onset (19). Patients with NIHSS scores as low as 4 points were included, although these patients represented a minority of the overall cohort. A subgroup analysis of the data suggested that patients with NIHSS scores < 10 may benefit from treatment when compared to placebo, but the study was underpowered to demonstrate a significant difference between these two groups (19). Further study to determine whether perfusion imaging can identify patients with mild stroke symptoms who might benefit from IV thrombolysis is warranted.

The WAKE-UP trial used MRI to identify stroke patients who are likely to benefit from IV thrombolysis when they present with an unknown time of symptom onset (20). Patients in this trial were enrolled if they had a mismatch between the ischemic core on DWI and corresponding hyperintense signal abnormality on FLAIR imaging, which suggests that their time from symptom onset is likely ≤4.5 h (after which FLAIR signal is typically hyperintense). WAKE-UP included patients with NIHSS scores as low as 4 as long as their symptoms were disabling. In a subgroup analysis, patients with NIHSS scores < 10 and disabling symptoms had significantly improved outcomes compared to placebo. These findings underscore that neuroimaging may be used to guide IV thrombolysis treatment decisions in patients with more mild stroke symptoms.

In summary, patients presenting with minor stroke severity (NIHSS scores ≤ 5) and disabling symptoms may still benefit from treatment with alteplase. The presence of a disabling neurologic deficit is a key feature in screening for eligibility and is an important adjunct to the NIHSS. Imaging plays a supportive role in the earlier time window patients but is more informative in late window patients when the amount of core infarct or time from symptom onset needs to be better characterized. Currently there is insufficient evidence supporting treatment of minor stroke patients with thrombolysis in later time windows but further studies are warranted.

## Minor Ischemic Stroke With Large Vessel Occlusion

Endovascular thrombectomy is a well-established treatment for acute ischemic stroke patients with NIHSS score ≥ 6 and concomitant large vessel occlusion (LVO) of the internal carotid artery (ICA) or the first part of the middle cerebral artery (MCA-M1) (5). Patients with minor stroke severity were not included in the landmark randomized thrombectomy trials that were reported between 2015 and 2018. As a result, there is a paucity of data to guide ET treatment decisions in patients with minor stroke symptoms due to LVO. Current ASA guidelines reflect this scarcity of high-level evidence and state that ET may be reasonable in patients with NIHSS < 6 (13). There are no specific comments about disabling symptoms such as those described in the IV thrombolysis literature and recommendations, which introduces additional uncertainty in the treatment of these patients.

**Figure 1:**
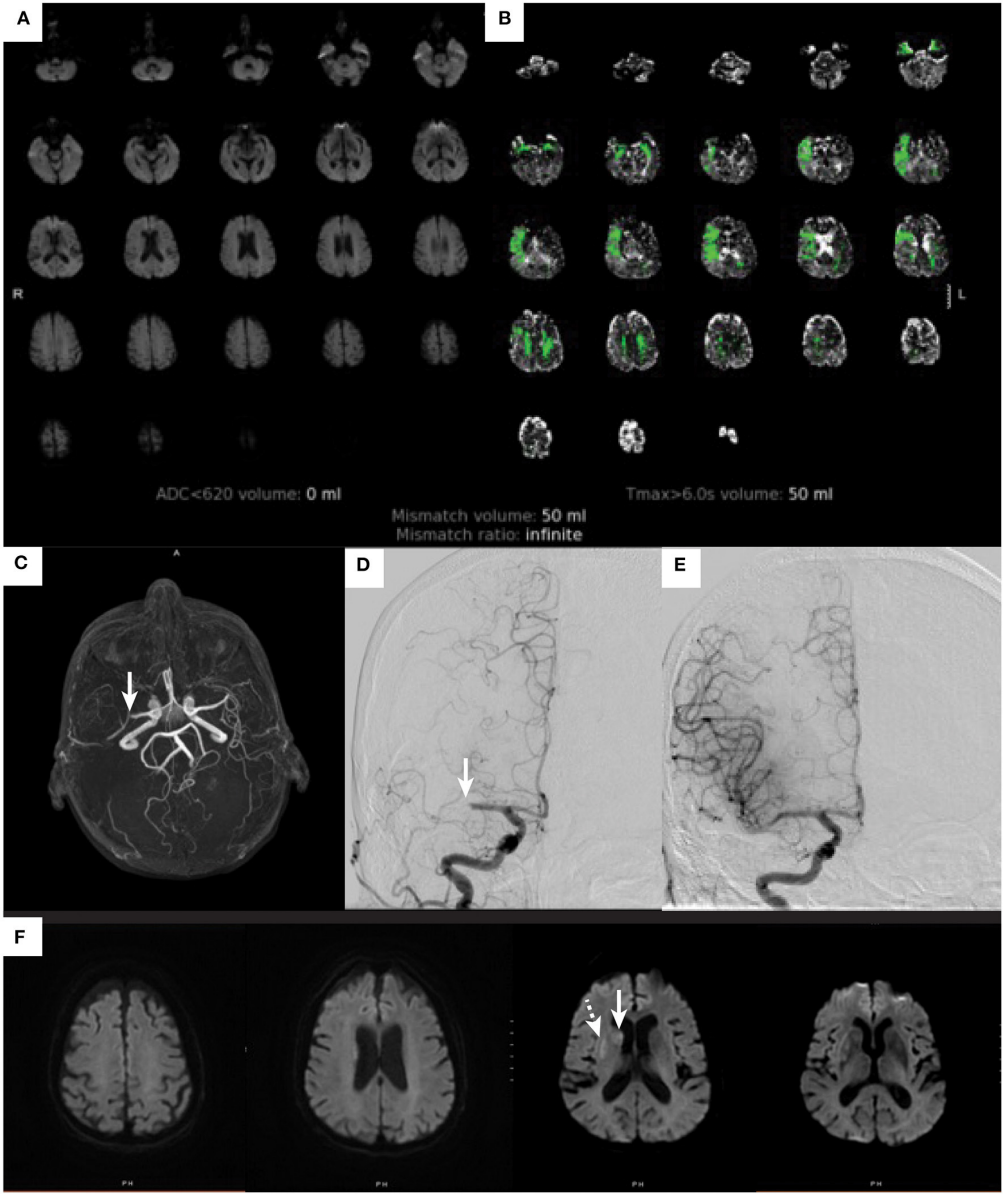
Ischemic stroke in an elderly patients with mild symptoms. An 81-year-old woman with atrial flutter on apixaban, hypertension, hyperlipidemia, and baseline modified Rankin score of 0 developed acute onset left facial droop with dysarthria and left hand weakness. These symptoms lasted for about 20 min and then completely resolved by the time she arrived to the emergency department. On initial examination she had an NIHSS score of 0. She was not treated with IV alteplase due to resolution of symptoms. A diffusion-weighted image **(A)** does not show any evidence of cerebral infarction (ADC < 620 volume 0), and perfusion imaging **(B)** demonstrates a perfusion deficit within the right MCA territory (Tmax > 6 s volume 50 ml). An MR angiogram [arrow **(C)**] shows a right M1-MCA occlusion. The patient underwent a cerebral angiogram that identified the right M1-MCA occlusion [arrow **(D)**], which was successfully treated by thrombectomy with complete revascularization **(E)**. A post thrombectomy diffusion-weighted MRI **(F)** demonstrates small cerebral infarctions within the caudate (arrow) and putamen (dashed arrow).

Given that the average NIHSS score of a patient with large vessel occlusion is 10 (21), one might question the frequency of patients with mild symptoms and LVO. In one study, about 13% of all acute ischemic stroke patients had an NIHSS of < 8 points and an LVO (22). Another study evaluated only patients with mild symptoms and found that within this group about 38% of patients had an LVO (23). Numerous other studies report varying percentages of mild ischemic stroke patients with LVO depending on the NIHSS cutoff (24, 25). These data illustrate that mild symptoms can be misleading and that there is a significant number of patients with low NIHSS scores who have a large vessel occlusion that would be amenable to thrombectomy treatment. One common cause for a patient to present with mild symptoms despite the presence of LVO is good collateral circulation that sustains the penumbra (tissue at-risk). It is important to recognize this subset of patients due to the potential for worse outcome should the collateral circulation collapse. Whether patients with mild stroke and LVO should be treated with endovascular thrombectomy remains highly debated and is a topic of ongoing randomized trials. An example case from our institution is shown in Figure 1.

## Mild Ischemic Stroke With Large Vessel Occlusion and Early Neurologic Deterioration

Strong collateral circulation often underlies mild ischemic stroke symptoms in the presence of an LVO. However, collaterals may reach a critical point and collapse with subsequent clinical worsening. This concept, termed early neurologic deterioration (END), describes worsening of stroke symptoms by four or more points on the NIHSS within 24 h of presentation and is not caused by intracranial hemorrhage (26). Recent data illustrated that approximately 12% of patients with mild stroke and LVO will progress to END despite treatment with alteplase (27). The vast majority who decline will do so within the first several hours of hospital presentation (28), which indicates that timely treatment is critical.

The risk of END is associated with several different factors. For example, the site of LVO has been well-described as a predictor of END in minor ischemic stroke. In one study, 30% of patients with occlusions involving the ICA terminus or tandem occlusion of the ICA and MCA-M1 suffered early deterioration. These patients had all been treated with intravenous thrombolysis as well (23). Patients with occlusions involving the ICA, ACA, MCA-M1, and basilar arteries were at least twice as likely to suffer early deterioration despite treatment with alteplase (23).

Another important factor associated with END is thrombus length. One study measured thrombus length on MRA, CT, or CTA and discovered that length is independently associated with END and the risk increases proportionately with increasing size (27). The investigators dichotomized length to demonstrate that thrombi measuring nine or more millimeters in size would yield three times greater odds of progressing to END (27). The authors suggested that larger thrombi were associated with END because early recanalization could not be achieved with IV thrombolysis alone. It has been previously demonstrated that patients with larger thrombi have suboptimal reperfusion rates after alteplase (29).

The potential for early neurologic deterioration in minor ischemic stroke patients with LVO poses a dilemma for providers. Although this pathway is overall uncommon, it may be more likely to occur with proximal cervical or cerebral artery occlusions and longer thrombi. If patients at risk of collateral circulation collapse and END could be accurately identified, these patients may be optimal to consider for thrombectomy treatment.

## Evaluating the Collateral Circulation in Minor Stroke With LVO

To date, there is no convincing association between collateral circulation and early neurologic deterioration. Some perfusion imaging parameters can be used as a surrogate for collateral circulation and are of interest in predicting END. Hypoperfused tissue with a disproportionately large amount of Tmax > 10 s delay compared to Tmax > 6 s delay is known to be associated with poor collateral circulation (30). One might speculate that patients with larger Tmax > 10 s volumes could be at risk for END. Saleem et al. (28) evaluated several different factors related to collateral circulation including perfusion-dependency of symptoms and Tmax perfusion volumes at thresholds of 6 and 10 s in a cohort of 122 patients, but none was independently associated with END. In a retrospective cohort of 81 patients with minor symptoms and LVO, Lee et al. (31) noted that patients who declined were significantly more likely to have larger baseline core and penumbra volumes on CT perfusion. These studies are limited by their small size and retrospective design.

## Outcomes in Patients With Minor Stroke and LVO

Data for outcomes in minor stroke treated with thrombectomy are limited to retrospective and observational cohorts. The most salient questions are (1) is EVT safe and feasible in this patient population and (2) is EVT more likely to yield improved outcomes compared to best medical therapy. A recent comprehensive meta-analysis published by McCarthy et al. included 24 different studies and found encouraging evidence for the overall safety of endovascular therapy (32). This finding is not surprising given that the technical aspects of the procedure would not differ among these patients and others with LVO. However, there were some negative aspects to treatment with ET in a cohort of patients from our center. Patients treated with ET had a longer length of stay and were more likely to be discharged to a skilled nursing facility, although there was no detectable impact on the rate of good outcome as measured by modified Rankin Scale score ≤ 2 (65% in medical group and 56% in ET group, *p* = 0.25). Due to the retrospective nature of the study it is possible that the ET group may have included sicker patients. There were also baseline differences between the groups with fewer patients receiving alteplase and more tandem occlusions in the ET group (33).

The second question regarding superiority of ET compared to best medical management in minor stroke with LVO is less clear. One of the largest studies to date was published by Dargazanli et al. and included a cohort of 301 patients. Half of these patients received best medical therapy and the other half received up-front ET along with best medical therapy. There was no significant difference in the rate of excellent or favorable outcome between the two groups (22). A second cohort of similar size, however, demonstrated a significant benefit of up-front ET with a rate of good outcome reaching 84% compared to 70% in the medical therapy group (34). Other studies have more specifically compared IV thrombolysis with EVT and found no difference in the rate of good outcome (35).

A final point to consider is delayed endovascular therapy. The above studies evaluated up-front ET, but it might be reasonable to offer best medical therapy first and follow up with endovascular therapy if neurologic deterioration occurs. Seners et al. (27) identified a subset of patients with minor ischemic stroke and large vessel occlusion who received alteplase and subsequently developed END. Just over half of the patients who deteriorated were selected for rescue ET and the vast majority achieved successful reperfusion (82%). Compared to patients who deteriorated and did not receive any ET, those who underwent ET were three times more likely to have a good outcome (27). In this cohort overall, the patients who suffered END had worse outcomes but this was mitigated to some degree with rescue thrombectomy.

In conclusion, studies have demonstrated reasonable safety and feasibility of ET for patients with minor ischemic stroke symptoms and concomitant LVO. Whether ET provides any additional benefit beyond best medical management is not clear. The “wait and treat” approach might be a reasonable alternative to up-front intervention, and data show that rescue thrombectomy may be beneficial in this situation. However, the opportunity for a good outcome may be diminished in the event of early neurologic deterioration regardless of rescue thrombectomy. Resource availability further complicates ET treatment decision-making process. When patients with LVO and mild stroke symptoms are monitored at a smaller community hospital with a “wait and treat” approach, further treatment delays may be incurred should the patient require transfer to a thrombectomy-capable center upon deterioration. If patients at risk for END could be accurately identified, then transfer to a tertiary stroke center could be quickly initiated. In our hospital, we often transfer such patients from community hospitals to our facility, where they are closely monitored and neurointerventional physicians are on-call for immediately treatment should clinical deterioration occur.

## Areas of Uncertainty and Need

**Figure 2:**
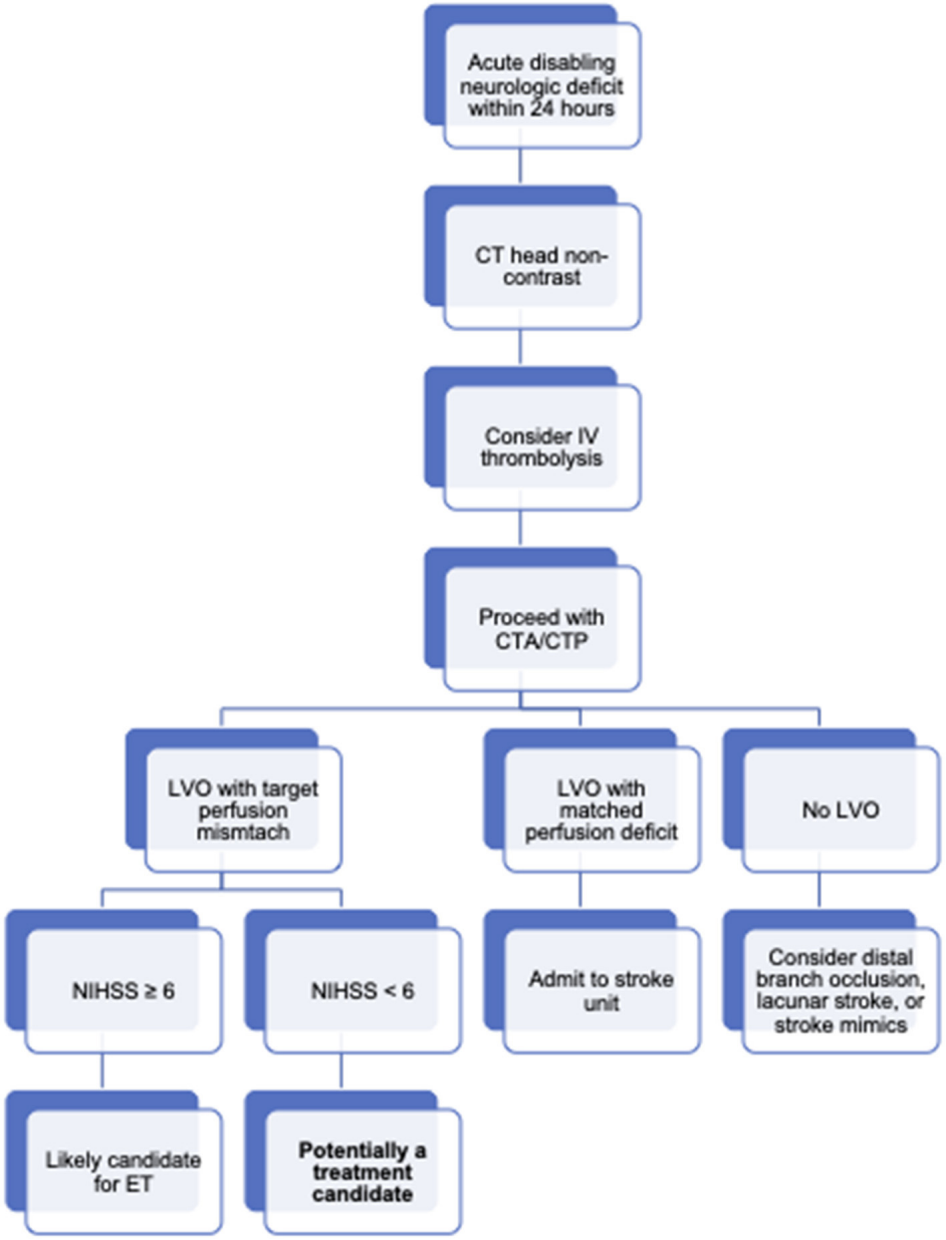
Treatment diagram for patients with a LVO and mild stroke symptoms. Board overview of workflow for acute ischemic stroke patients at our institution. In general, patients with an acute neurologic deficit (even those scoring low on the NIHSS) will be taken for non-contrast head CT if stroke is clinically suspected. In the absence of intracranial hemorrhage or early ischemic changes, patients may be considered for thrombolysis if symptoms remain diabling and the patient is within 4.5 hours of symptoms onset. Extended window thrombolysis is considered on a case-by-case basis and is not depicted here. Patients treated with thrombolysis and patients not treated with thrombolysis but still suspected to have an acute stroke clinically are further imaged with CT angiography and perfusion. Identification of an LVO and a target perfusion mismatch profile (CBF < 30% volume of < 70 cc, mismatch ratio ≥ 1.8, mismatch volume ≥ 15 cc) leads to activation of the stroke interventional team. Patients with NIHSS scores of 6 or more points are usually treated with ET. Patients with NIHSS < 6 are considered.

The ability to predict clinical decline is a critical factor in decision making for patients with minor stroke symptoms and LVO. Patients are likely to do well with best medical therapy unless they develop early neurologic deterioration, at which point they may be at risk for poor outcome regardless of rescue treatment. Perhaps patient selection for treatment should be performed in a manner similar to late-window thrombectomy where perfusion imaging plays an important role in selection for patients presenting 6–24 h from symptom onset (Figure 2) (3, 4). Both groups share an underlying pathophysiology of LVO with peri-ischemic tissue sustained by strong collateral circulation. With this idea in mind, some of the techniques used for late-window thrombectomy selection, such as perfusion imaging, could be shared in selecting patients with minor symptoms and LVO for ET.

As discussed earlier, thrombus length and location are some established predictors of END. There are other advanced imaging measures that could identify patients at risk of decline. For example, patients with more critically impaired perfusion at presentation could be considered high risk. Hypoperfusion severity could be measured by Tmax > 10 s volume or the hypoperfusion intensity ratio (HIR). Higher HIR values are known to be correlated with more rapid infarct progression (36, 37).

Grading the collateral circulation is another potential way to identify patients at risk for clinical decline. There are numerous collateral scoring systems that exist, each with strengths and weaknesses. The gold standard for rating pial collaterals is digital subtraction angiography (DSA). However, this technique is the most invasive method for collateral assessment, which renders it a poor screening tool. Single-phase CTA captures arterial filling over time after a bolus injection, while the more advanced multi-phase CTA characterizes blood flow in the arterial, peak venous, and late venous phases. Multi-phase CTA may provide a more nuanced evaluation of the collateral circulation and is validated to predict outcomes in acute ischemic stroke (38). Most patients presenting with acute ischemic stroke will undergo CTA as part of the initial diagnostic workup. Further study is needed to determine if CTA can serve a dual purpose as a screening tool for later decompensation. In DEFUSE 3, patients with good collateral scores graded by the Tan/Maas scales with single-phase CTA had smaller ischemic core volumes and decreased core volume growth (37). This subset of late-window patients could be similar to those with minor symptoms and LVO with strong collateral circulation.

Other studies have compared CTA-based collateral scoring with CT perfusion imaging for selecting patients who would most likely benefit from endovascular therapy. The two methods have similar capability of predicting outcomes in a late-window cohort (38) but these techniques have not been thoroughly explored in patients with LVO and minor stroke symptoms.

## Current Clinical Trials

There are a number of trials investigating best management strategies for acute ischemic stroke with low NIHSS. The ENDOLOW study is currently recruiting patients with NIHSS 0–5 and objective neurological deficits who present within 8 h of symptom onset to be randomized to best medical therapy or ET. This trial requires imaging confirmation of an LVO (ICA, MCA M1, or proximal M2) and absence of a large core infarct judged by ASPECTS ≥ 6 or estimated ischemic core volume of <70 ml (determined by CT perfusion imaging as a CBF < 30% reduction). Crossover from the medical group to the endovascular group is permitted in the event of neurologic deterioration (Clinicaltrials.gov study number NCT04167527). The study design echoes that of late-window thrombectomy trials that relied on advanced imaging to identify the amount of salvageable tissue.

A second clinical trial, MOSTE, is currently enrolling in Europe. This study is randomizing patients with NIHSS < 6 or clinical stroke symptoms within 24 h of last known well to receive either best medical therapy or ET. Imaging criteria is broad and allows for patients with ASPECTS ≥ 6 with a confirmed LVO involving the ICA, MCA (M1 or M2 segments) (Clinicaltrials.gov study number NCT03796468). This study will likely capture a more heterogeneous patient population compared to the ENDOLOW study, but both studies will provide meaningful data for selecting patients for endovascular treatment.

Third, the TEMPO-2 study focuses on medical management in patients with NIHSS ≤ 5. Patients in TEMPO-2 are randomized within 12 h of symptom onset to either Tenecteplase or antiplatelet therapy. Uniquely, this study includes patients with transient ischemic attack in addition to patients with ongoing symptoms at the time of enrollment. Imaging requirements for enrollment include multi-phase CTA to determine the presence of complete or near-complete occlusion of any identifiable vessel supplying anterior or posterior circulations, or evidence of a focal perfusion abnormality that can be correlated with symptoms (Clinicaltrials.gov study number NCT02398656). This study takes a novel approach by favoring thrombolytic treatment based on imaging findings of LVO or perfusion changes. Inclusion of patients with no symptoms at all will provide an interesting view on treatment selection in this population.

Of note, none of these clinical trials has emphasized the need for disabling neurologic deficits. The presence of any deficit is considered meaningful in the context of a corresponding LVO, and the presence of a LVO is required for enrollment in each of these studies. Imaging clearly plays a larger role in screening for patients with low NIHSS who would still benefit from treatment.

## Conclusions

Patients who present with acute ischemic stroke and low NIHSS are an important subgroup of the broader ischemic stroke population. Treatment decisions for IV thrombolysis and ET are different but rely on shared principles. The presence of disabling symptoms is a cornerstone consideration for both intravenous and endovascular therapy and would likely warrant treatment with alteplase at a minimum. On the other hand, patients with non-disabling symptoms are likely to do well without interventions. The benefit of endovascular therapy up-front has not been definitively established in patients with minor symptoms and large vessel occlusion. In the event of clinical decline, rescue thrombectomy may yield improved outcomes. Clinical trials are needed to understand the value of endovascular therapy in this patient population, and identification of patients likely to develop END would further aid in optimizing patient selection.

## Author Contributions

DS prepared the manuscript and figure. JH critically reviewed the manuscript and figure. Both authors contributed to the article and approved the submitted version.

## Conflict of Interest

JH was the consultant for Medtronic and Microvention and member of the Medical and Scientific Advisory Board, iSchemaView. The remaining author declares that the research was conducted in the absence of any commercial or financial relationships that could be construed as a potential conflict of interest.

## Publisher's Note

All claims expressed in this article are solely those of the authors and do not necessarily represent those of their affiliated organizations, or those of the publisher, the editors and the reviewers. Any product that may be evaluated in this article, or claim that may be made by its manufacturer, is not guaranteed or endorsed by the publisher.
